# Cell migration on material-driven fibronectin microenvironments†
†Electronic supplementary information (ESI) available. See DOI: 10.1039/c7bm00333a. All the original data related to this article are within the depository of the University of Glasgow with DOI: 10.5525/gla.researchdata.419


**DOI:** 10.1039/c7bm00333a

**Published:** 2017-06-06

**Authors:** E. Grigoriou, M. Cantini, M. J. Dalby, A. Petersen, M. Salmeron-Sanchez

**Affiliations:** a Division of Biomedical Engineering , School of Engineering , University of Glasgow , Glasgow , UK . Email: Manuel.Salmeron-Sanchez@glasgow.ac.uk; b Centre for Cell Engineering , University of Glasgow , UK; c Berlin Brandenburg Center for Regenerative Therapies , Charité-Universitätsmedizin Berlin , Berlin , Germany . Email: Ansgar.Petersen@charite.de

## Abstract

Cell migration is a fundamental process involved in a wide range of biological phenomena. However, how the underlying mechanisms that control migration are orchestrated is not fully understood. In this work, we explore the migratory characteristics of human fibroblasts using different organisations of fibronectin (FN) triggered by two chemically similar surfaces, poly(ethyl acrylate) (PEA) and poly(methyl acrylate) (PMA); cell migration is mediated *via* an intermediate layer of fibronectin (FN). FN is organised into nanonetworks upon simple adsorption on PEA whereas a globular conformation is observed on PMA. We studied cell speed over the course of 24 h and the morphology of focal adhesions in terms of area and length. Additionally, we analysed the amount of cell-secreted FN as well as FN remodelling. Velocity of human fibroblasts was found to exhibit a biphasic behaviour on PEA, whereas it remained fairly constant on PMA. FA analysis revealed more mature focal adhesions on PEA over time contrary to smaller FAs found on PMA. Finally, human fibroblasts seemed to remodel adsorbed FN more on PMA than on PEA. Overall, these results indicate that the cell–protein–material interface affects cell migratory behaviour. Analysis of FAs together with FN secretion and remodelling were associated with differences in cell velocity providing insights into the factors that can modulate cell motility.

## Abstract 2

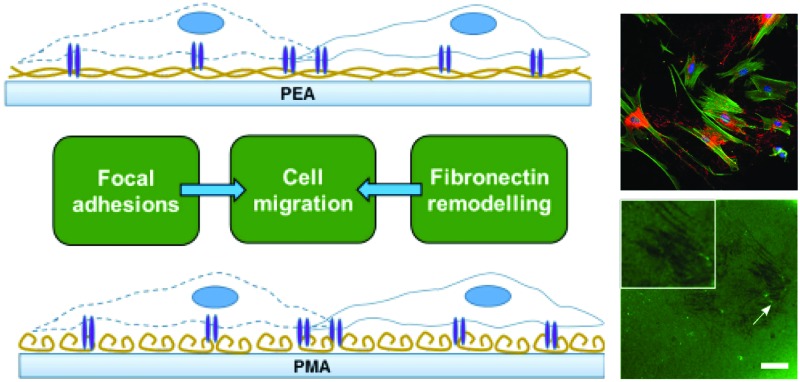
Cell migration depends on the physical state of fibronectin, fibrillar *vs.* globular, which can be controlled by engineering biomaterials.

## Introduction

Cell migration has a central role in physiological events and in pathologies such as embryonic development, immune response and cancer metastasis. It is a highly regulated, cell-specific process characterised by a complex interplay between the cell and the extracellular matrix (ECM). Cell locomotion involves polarisation and protrusion at the leading edge and these events are dependent on actin polymerisation and cell attachment to the ECM. These events lead to the transmission of traction forces which facilitate cell translocation and retraction of the rear.^1^,^2^

Adhesion to the ECM and signal transduction are mediated *via* focal adhesions (FAs), which are diverse protein complexes.^3^ FAs physically link the actin cytoskeleton to the ECM primarily through integrins which are their main trans-membrane heterodimer cell receptors, but also through mechanotransductive and signalling pathways comprising *e.g.* focal adhesion kinase (FAK) and vinculin.^4^ During cell migration, repeated cycles of FA assembly and disassembly occur, followed by a rapid change in their protein composition over time in response to external cues.^4^–^6^ It has been speculated that FA characteristics such as size and morphology determine cell migration.^7^ Also, it has been shown that varying the size of FAs using nanopatterned surfaces correlates with specific cell migratory behaviour.^8^ Additionally, several studies have demonstrated the relationship between cell motility and the chemical and physical properties of the substrate. The variables reported to impact cell motility are the ligand density presented by the substrate, the integrin expression levels of the cells and the integrin–ligand affinity.^9^,^10^ It is also interesting to note that many studies reported maximum cell speed in intermediate levels of cell-substratum adhesiveness.^9^–^11^

Fibronectin (FN) is the main component of the ECM and it modulates several cell processes, such as adhesion, differentiation and proliferation. It is a 440 kDa dimeric glycoprotein found in insoluble and soluble forms. Each subunit contains three types of repeating units (FN type I, II and III) and consists of multiple recognition sites that bind other ECM and FN molecules, growth factors and cell receptors, such as integrins.^12^ It has been found that optimal cell migration requires growth factors^13^,^14^ as well as receptor binding domains.^15^

Despite significant progress, it is still challenging to fully understand the molecular mechanisms coordinating cell migration. It is well established that cells respond to external stimuli, for example to mechanochemical signals and to the stiffness and dimensionality of the ECM.^16^,^17^ Thus, it is today well accepted that biomaterials provide a way to alter cell motility in a controlled manner *via* the modulation of physico-chemical material parameters. However, much less is known about how biomaterials influence cell behaviour indirectly, *via* the modulation of ECM protein adhesion and conformation. In this regard, cell migration experiments have been conducted implementing surfaces coated with ECM proteins aimed at mimicking the properties of the *in vivo* matrix. FN is an excellent candidate due to its high sensitivity to cell-derived strain resulting in major conformational changes.^18^,^19^ Additionally, studies have shown that the organisation and activity of adsorbed FN is regulated by the chemical properties of the underlying substrate.^20^–^22^

**Fig. 1:**
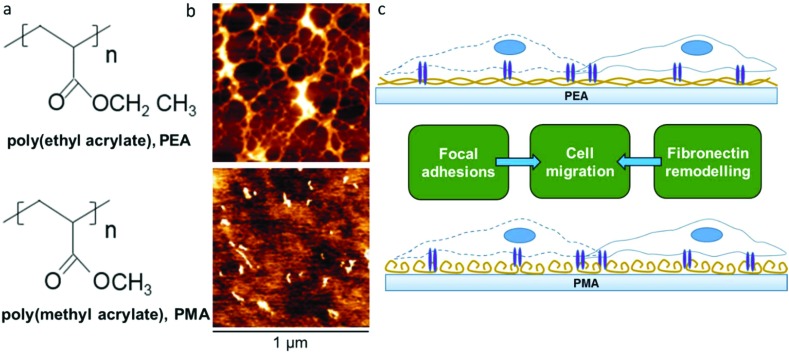
Chemical structure of PEA and PMA (a). Fibronectin distribution on PEA and PMA as observed with the height magnitude in the AFM. The protein was adsorbed for 10 min from a solution of concentration 20 μg ml^–1^ (b). Schematic representation of cell migration on PEA and PMA coated with fibronectin (c).

We have previously shown that poly(ethyl acrylate) (PEA) surfaces trigger the formation of a physiological-like network of adsorbed FN – material driven FN nanonetworks. However, the same phenomenon is not observed on poly(methyl acrylate) (PMA), on which FN molecules organise into globular aggregates upon adsorption.^23^ This PMA/PEA system thus provides an excellent pair of chemically and physically similar controls that either do or don't drive FN fibrillogenesis. This new work aims at exploring the migrational behaviour of human fibroblasts on these FN coated surfaces (Fig. 1). Migration experiments carried out over the course of 24 hours showed clear differences in cell speed that motivated a closer look at the relationship between FN conformation, cell adhesion, matrix secretion, matrix reorganisation and cell migration.

## Materials and methods

### Preparation of samples

PEA and PMA polymers were synthetized by radical polymerization of ethyl acrylate and methyl acrylate. The initiator was benzoin at 1 wt% and 0.35 wt%, respectively. The molecular weight for both polymers was measured by GPC (*M*_w_ 600 kDa, *M*_n_ 90 kDa). Samples were then dried by vacuum extraction to constant weight and solubilized in toluene 2.5% w/v for PEA and 6% w/v for PMA. In order to obtain a thin layer of polymer, spin coating was used to coat 12 mm-diameter glass coverslips with PEA and PMA solutions for 30 s at a velocity of 2000 and 3000 rpm respectively. Then, samples were dried at 60 °C in vacuum for 2 h to remove the excess toluene.

### Atomic force microscopy

Scanning of the surfaces was performed using tapping mode atomic force microscopy (AFM; Nanowizard 3 from JPK). Cantilevers (Bruker) with a force constant of 3 N m^–1^ and a resonance frequency of 75 kHz were used.

### Protein adsorption

Human plasma FN (Sigma) solution in Dulbecco's phosphate buffered saline (PBS) at 20 μg ml^–1^ was coated onto UV-sterilised PEA and PMA coverslips for 1 h.

### Cell isolation and cell culture

Primary human dermal fibroblasts isolated from skin biopsies were used in this study (25 years old male donor). For fibroblast isolation, the epidermal layer and fat tissue were removed and dermis pieces were distributed on the bottom of T75 cell culture flask. Dermis was allowed to attach to the culture bottle by incubation without culture medium for 1 h (37 °C, 5% CO_2_). Subsequently, cell culture expansion medium was carefully added avoiding floating of skin pieces. During subsequent culture, medium was exchanged every 2–3 days. After 2–3 weeks, outgrowth of fibroblasts from the dermis was observed. At 30–50% confluency, dermis pieces were removed. Adherent cells were trypsinized and transferred to a new cell culture flask. Fibroblasts were grown into full confluency twice in cell culture flasks of increasing size (T175, T300) to obtain a pure fibroblast culture without keratinocytes. Fibroblasts were cryopreserved in passage 3–4. For experiments, cells were thawed and cultured until 70–80% confluency in Dulbecco's modified Eagle's medium (4500 mg per l glucose) supplemented with 1% P/S and 10% FBS. Cells were trypsinized and brought into suspension at the desired concentration for subsequent cell culture experiments.

### Migration assay

PEA and PMA coverslips were coated with FN at 20 μg ml^–1^ (Sigma) for 1 h. Cells were harvested by trypsinization and then seeded on the surfaces at density of 5000 cells per cm^2^. Expansion medium was used for cell dilution. The samples were incubated at 37 °C for 3 h to allow initial attachment. Afterwards, nuclear staining was carried out by incubating the cells with Hoechst® 33342 nucleic acid dye for 10–15 min at 37 °C. Next, the medium was replaced by fresh cell culture medium and the plate was mounted in the motorised staged of a Leica DMI6000 timelaps microscope to record cell migration. Four ROIs in each sample were selected and images were recorded every 10 min for 24 h. Quantification of velocity was carried out using the software Volocity. Four time-lapse videos were analysed per condition by automatically tracking the cell nucleus.

### Immunostaining of focal adhesions

PMA and PEA coverslips were coated with FN at 20 μg ml^–1^ (Sigma) for 1 h. Samples were washed with PBS and seeded with cells at a density of 5000 cells per cm^2^. Cells were cultured at 37 °C for 6 h and 22 h respectively. After the given time, cells were fixed with 4% formaldehyde for 30 min at 4 °C and washed again in PBS. Next, samples were permeabilised for 5 min at 4 °C (0.5% v/v Triton X-100, 10.3% w/v saccharose, 0.292% w/v NaCl, 0.06% w/v MgCl_2_, and 0.476% w/v HEPES adjusted to pH 7.2) and incubated with 1% w/v BSA/PBS solution for 5 min at 37 °C. Next, samples were stained using primary monoclonal antibody against vinculin (Sigma-Aldrich) overnight 4 °C. The next day, samples were washed three times (DPBS/Tween-20 0.5%) for 5 min and were stained using secondary biotinylated anti-mouse antibody (Vector Laboratories) for 1 h at 37 °C. Samples were washed again and incubated with streptavidin-FITC (Vector Laboratories, Inc.) and phalloidin-rhodamine for 30 min at 37 °C in dark. Finally, cells were washed and mounted with Vectashield containing DAPI (Vector Laboratories, Inc.). Images were taken with an inverted fluorescent microscope (Zeiss AXIO Observer Z1). Data about the size and length of focal adhesions were obtained by using an online focal adhesion analysis server.^24^

### Fibronectin secretion

PMA and PEA coverslips were coated with FN at 20 μg ml^–1^ for 1 h. Human fibroblasts were seeded at a density of 5000 cells per cm^2^ and incubated at 37 °C for 6 h and 22 h. Medium supplemented with 10% FBS was used. Samples were fixed with formaldehyde 4% at 4 °C for 30 min and next they were incubated with permeabilisation buffer for 5 min and blocked (1% v/v BSA/PBS) for 30 min. Next, samples were stained using primary monoclonal antibody against cellular FN (Abcam) for 1 h at room temperature. After they were washed (0.5% v/v Tween20/PBS), they were incubated with secondary Cy-3 anti-mouse antibody (Jackson ImmunoResearch) together with Alexa fluor 350 phalloidin (Invitrogen) for 1 h. Finally, samples were washed again and they were mounted using Vectashield without DAPI (Vector Laboratories, Inc.). Images were taken with an inverted fluorescent microscope (Zeiss AXIO Observer Z1) and image analysis was carried out using ImageJ.

### Fibronectin reorganisation

PEA and PMA cover slips were coated with FN conjugated with FITC at 20 μg ml^–1^ for 1 h according to an established protocol.^25^ Human fibroblasts were seeded at a density of 5000 cells per cm^2^. Medium supplemented with 10% FBS was used for the seeding. Cell culture was maintained at 37 °C for 6 h and 22 h. Next, cells were fixed with 4% formaldehyde for 30 min at 4 °C. After washing with PBS, cells were incubated with permeabilisation buffer for 5 min and then they were blocked (1% v/v BSA/PBS) for 30 min. Afterwards, cells were stained with primary polyclonal antibody against cellular FN (Abcam) for 1 h. Next, samples were washed (0.5% v/v Tween20/PBS) and they were stained with secondary Cy-3 anti-mouse antibody (Jackson ImmunoResearch) and Alexa Fluor 350 phalloidin for 1 h. Finally, samples were mounted with Vectashield without DAPI (Vector Laboratories, Inc.). Images were taken with an inverted fluorescent microscope (Zeiss AXIO Observer Z1). Image analysis was carried out using ImageJ. Actin was used as mask for the reorganisation of the FITC-labeled FN. The mean intensity of FITC-labeled FN underneath the cell was normalised to the mean intensity of the area outside of the cell.

### Statistical analysis

One-way ANOVA using a Bonferroni *post hoc* test were used to establish significant differences *p < 0.05, **p < 0.01, ***p < 0.0001 (GraphPad Prism software). Data are expressed as mean ± standard deviation.

## Results

### Fibronectin adsorption

PMA and PEA are chemically very similar, with PEA containing one more methyl group in the lateral chain than PMA (Fig. 1a). However, while similar, the distribution of FN upon adsorption on PEA and PMA is very different as has been previously demonstrated^23^ and as we confirm here using AFM. Fig. 1b shows AFM height images of PEA (Fig. 1b top) and PMA (Fig. 1b bottom) coated with FN from solution concentration of 20 μg mL^–1^. The protein acquires a network-like fibrillar conformation on PEA whereas it is organised into globular aggregates on PMA. This difference in protein conformation is maintained also after incubation with DMEM supplemented with 10% FBS (cell culture conditions, Fig. S1†). This provides a simple approach to creating different environments on which to study cellular migration (Fig. 1c).

### Cell migration on protein coated surfaces

**Fig. 2:**
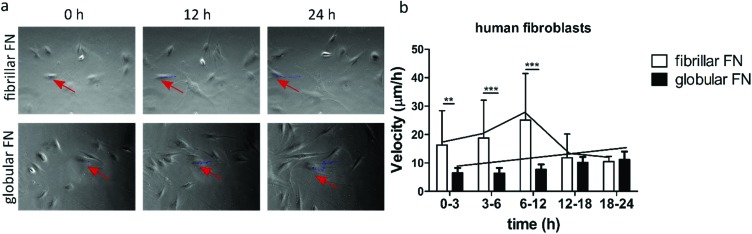
Characterization of cell migration on fibrillar (on PEA) and globular (on PMA) FN over the course of 24 h. Phase contrast pictures of human fibroblasts (a) on fibrillar FN and globular FN 0 h, 12 h, 24 h after attachment. Red arrows indicate the migration of a single cell over time. Velocity (μm h^–1^) of human fibroblasts (b). Videos of migratory cells were quantified to build the migration graphs (ESI Video 1†).

Cell migratory behaviour was studied on protein-coated surfaces (Fig. 2, Video S1†). Cell velocity (μm h^–1^) was analysed over the course of 24 h on protein coated PEA and PMA after 3 hours of initial attachment. Human fibroblasts (Fig. 2) moved rapidly on fibrillar PEA for the first 12 h and after this cell speed decreased. In contrast, no such initial rapid cell movement was observed on globular PMA, on which fibroblasts only slightly increased their speed over time. Cell velocity was highly significantly (*p* < 0.01) greater on fibrillar PEA than on globular PMA during the first 12 h, achieving up to ∼24 μm per hour on PEA compared to ∼10 μm per hour on PMA. At the later timepoints, (*t* > 12 h) cell migration speed on fibrillar FN (PEA) decreased strongly towards the level observed for globular FN (PMA).

### Focal adhesion analysis

**Fig. 3:**
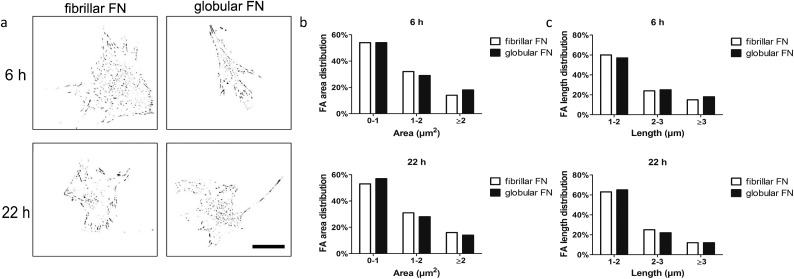
Characterisation of focal adhesions of human fibroblasts. Representative inverted binary images of focal adhesions on fibrillary (on PEA) and globular (on PMA) FN (a). Area and length distribution of focal adhesions of human fibroblasts on fibrillary (PEA) and globular (PMA) FN 6 h (b) and 22 h (c) after seeding. Scale bar: 50 μm.

Analysis of focal adhesions aimed at investigating whether there is a functional relationship between focal adhesion assembly and cell migration in dependence on the organisation of FN (fibrillar on PEA *vs.* globular on PMA) on the material interfaces. Fibroblasts were seeded on FN-coated PEA and PMA and vinculin staining was carried out after 6 h and 22 h of culture. Area and length distribution of focal adhesions were obtained by quantifying fluorescent images (Fig. 3).

Gross cell morphological differences were not observed between the samples and cells looked similarly well spread (Fig. S2†). For quantitative analysis of focal adhesions, we classified them based on area as immature (0–1 μm^2^), intermediate (1–2 μm^2^) and mature (>2 μm^2^) and further sub-classified by length as short (1–2 μm), intermediate (2–3 μm) and long (>3 μm). Analysis of the adhesions revealed skewed distribution (towards smaller adhesion as expected) on both fibrillar FN on PEA and globular FN on PMA (Fig. S3†). The fraction of mature FAs (≥2 μm^2^) remained constant with time on fibrillar FN (from 14% to 16%) and globular FN (from 15% to 14%) (Fig. 3 and Fig. S4, S5†). It is notable that the fraction of short FAs (<2 μm) increased on PMA over time more than on PEA (Fig. 3b and c). Cell size decreased after 22 h on fibrillar FN, while no changes were observed for globular FN on PMA (Fig. S5a†).

### Fibronectin secretion

**Fig. 4:**
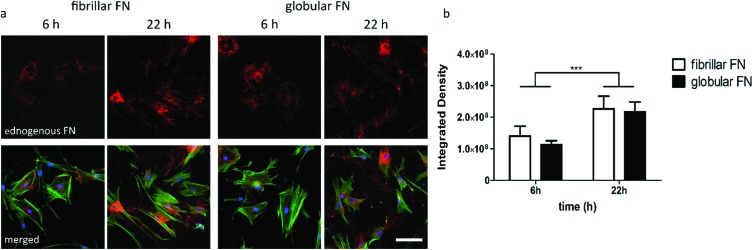
Fluorescent images of human fibroblasts (a) on fibrillar (PEA) and globular (PMA) FN 6 h and 22 h after seeding. Staining of cellular FN (red), actin (green) and nuclei (blue). Scale bar is 100 μm. Quantification of FN secreted by human fibroblasts (b) on fibrillar and globular FN (white and black bars respectively) 6 h and 22 h after seeding.

As a next step, the effect of PEA/PMA engineered FN morphology on cell secreted, endogenous, FN was explored. Cells were seeded on FN coated PEA and PMA and staining of endogenous FN was carried out 6 h and 22 h after seeding. Immunofluorescent staining of fibroblasts showed an increase of deposited endogenous FN over time (Fig. 4a) confirmed by quantification (Fig. 4b).

### FN remodeling

**Fig. 5:**
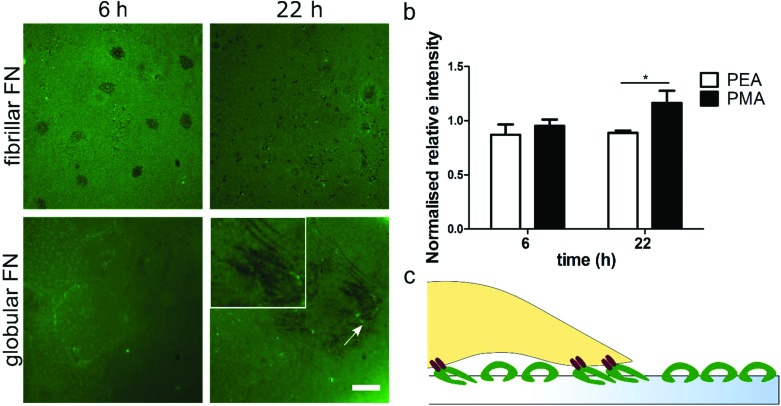
FN reorganisation. Fluorescent pictures of FITC-labelled FN on PEA (fibrillar) and PMA (globular) after reorganisation by human fibroblasts for 6 h and 22 h. Scale bar is 30 μm (a). Normalised fluorescence intensity of FN within the cell compared with the intensity outside the cell area (b). Schematic representation of cell-mediated reorganisation of FN. 2 h.

FITC-labelled FN was used in order to assess how fibroblasts reorganise the pre-adsorbed layer of either globular (PMA) or fibrillar (PEA) FN over time. FN reorganization appears as dark areas against the fluorescent background, surrounded by brighter fibrils (Fig. 5). Image quantification showed that human fibroblasts were able to reorganise the adsorbed globular FN more effectively on PMA over time; this is demonstrated by the larger dark areas observed on PMA 22 h after seeding. On the other hand, no statistically significant differences were found in FN reorganisation on fibrillar FN on PEA over time (Fig. 5b). In addition, changes in the layer of FN were not observed in the absence of cells (Fig. S6†).

## Discussion

The ECM is a complex network that plays a fundamental role in biological processes. It provides not only structural support but also mediates mechanochemical cues.^26^ Mimicking the *in vivo* cellular complexity and the mechanisms governing cell–ECM interactions is critical in order to understand events for important cell functions such as cell motility. Cell/substrate interaction starts with cell adhesion to peptide motifs in ECM proteins (*e.g.* absorbed to biomaterials). This attachment allows cells to interact with their environment's properties and modulate their adhesion and migration.^27^ For example, it has been suggested that cells migrate towards stiffer substrates in a process called durotaxis.^28^,^29^

In this work, we investigated the migratory characteristics of human fibroblasts using two polymer surfaces with similar chemistries, PEA and PMA. Both polymers consist of a vinyl chain with a side chain that differs by only one methyl group (Fig. 1a). PEA and PMA have similar wettability and, when coated with FN, similar amount of the adsorbed protein has been measured.^30^ However, interestingly, FN undergoes different structural changes upon passive adsorption on PEA and PMA triggered by the chemical properties of the substrate.^31^ A network-like conformation is formed on PEA, whereas globular aggregates are observed on PMA (Fig. 1b). It has been reported that the different conformation of FN results in changes in the bioactivity of the molecule and in the availability of important cell binding domains.^20^,^23^,^30^,^32^

Cell migration is regulated by the ligand density, the ligand–integrin binding affinity and the integrin expression level.^11^ With this in mind, we explored whether FN conformation affects cell motility. Cell migration experiments showed that the initial speed of human fibroblasts was higher on the fibrillar networks assembled on PEA compared to globular FN on PMA. In contrast, cell speed (albeit slower) was maintained on PMA over time (Fig. 2b). Such biphasic behaviour (initial fast cell migration followed by speeds slowing down) has been previously described and these studies have cited that intermediate levels of cell adhesion were required for this enhanced migration to be seen.^9^,^10^,^33^ It is also known that multiple domains of FN contribute to cell migration *e.g.* RGD and the PHSRN synergy sequence,^15^,^34^,^35^ thus different structural patterns (*e.g.* globular and fibrillar) of the protein might result in different migratory characteristics. We have previously shown that the availability to cells of the PHSRN sequence, in a loop region of the 9^th^ type III repeat of FN, and of the growth factor binding domain, in the 12^th^–14^th^ type III of FN, to is higher on PEA than on PMA.^30^,^36^ Other studies have associated the PHSRN domain with enhanced cell migration *in vitro* and accelerated wound healing *in vivo*.^37^,^38^ In another work, increased migration and proliferation were induced when cells were stimulated with growth factors delivered with recombinant-linked FN fragments containing the type III 9–10/12–14 domains.^39^ Conformational changes of FN affect integrin binding resulting in altered cell proliferation and differentiation.^40^ In addition, high directional persistence of fibroblasts has been shown to require the engagement of both α_v_β_3_ and α_5_β_1_.^41^ We thus postulate that the network-like conformation of fibrillar FN on PEA provides a sufficient level of adhesiveness for enhanced fibroblast motility. This might be due to the more efficient presentation of important binding sites which allow better integrin-mediated substrate engagement. In contrast, the globular conformation of FN on PMA might alter the extent to which important binding domains are displayed, resulting in decreased cell motility compared to PEA.

Focal adhesions formation was thus assessed to give further insights into this process. Previous work did in fact correlate FA size and cell speed.^42^ In our study, it was interesting to see that adhesion size distribution was not dramatically different in fibroblasts on fibrillar or globular FN (Fig. 3b, c and Fig. S3, S4†). Thus, it appears that FA size, *per se*, was not responsible for cell migration on the substrates.

It has been shown that the ECM synthesised and secreted by cells can cause changes in cell migration.^43^ To further explore this related to cell motility, we then investigated whether cell migration was associated with cell-mediated FN remodelling, including its reorganisation and deposition. An increase in FN secreted on both surfaces (Fig. 4a) was observed over the course of 22 hours. However, no significant differences were found between substrates (Fig. 4b).

ECM remodelling induced by cell-generated forces is a fundamental and highly controlled process that regulates important cellular functions including cell migration. Previous work using 3D collagen matrices has, for example, reported higher invasiveness of breast cancer cells on areas that were not reorganised.^44^ Additionally, higher migration and invasion of breast cancer cells was found when ECM acquired a specific alignment.^45^ Thus, we explored the reorganisation of adsorbed FN by human fibroblasts in order to gain further insight on the different migratory response to FN conformation. FN remodelling was higher on globular FN, while on fibrillar FN no changes were seen (Fig. 5); this is comparable to previous reports from our group.^25^ Cells reorganized labelled globular FN over time, while no substantial changes were observed on fibrillar FN organised into nanonetworks on PEA. This may be due to the cells having to reorganise globular FN to be able to use the RGD and PHSRN groups efficiently while fibrillar FN is presented to the cells in a more immediately usable conformation. This lack of having to reorganise the adsorbed FN, we postulate, leads to the higher cell speeds observed on fibrillar FN. It could be further postulated that as cells tend to modify their surrounding environment before they secrete their own matrix^46^,^47^ and the strong interaction of FN with PEA (protein–material interaction) inhibits this matrix remodelling. This eventually results in a decrease in cell speed after the initial rapid migration phase.

In conclusion, during the early stages of attachment and migration, cells are in contact with the adsorbed layer of FN. The fibrillar conformation of FN on PEA allows for better cell attachment, which leads to increased migration speed. Over time, cells start to interact with the endogenous FN that they secrete and assemble as well as to remodel the underlying FN layer; these phenomena in turn affect their motility. We propose that the difference in cell interaction with the fibrillar and globular FN molecules causes the cells to migrate differently. For globular FN on PMA the cells have to reorganise FN before they can fully exploit it and by the time they have reorganised it they have changed from migratory/proliferative activity to a more matrix-secreting, differentiating activity; thus their speed is always slow. On fibrillar FN on PEA, on the other hand, however, they can immediately exploit the networks for movement and growth; this slows as they secrete their own matrix proteins and fail to reorganise the adsorbed exogenous FN on the PEA causing an initial speedy migration followed by a slow migration.

## Conclusions

Gaining better insights into the role of ECM in cell behaviour is fundamental in designing biomaterials that are able to better control cell response. Taking into consideration that ECM proteins are commonly used to functionalise biomaterials, it is of great importance to understand how FN conformation can direct cell fate. Previous studies have demonstrated the role of FN in cellular processes. Conformational changes of FN characterised by unfolding and stretching of fibrils have been observed over time and were associated with maturation and aging.^48^ FN has been found to undergo structural changes in pathological conditions such as cancer. FN unfolding was induced by soluble factors secreted by breast cancer cells.^49^ Similarly, cells exposed to tumour associated factors promoted FN unfolding.^50^

This work demonstrates that the material interface itself affects cell migration through conformational changes of adsorbed FN and cell-mediated FN reorganisation. Fibrillar FN organised into nanonetworks induced higher cell speed compared to globular FN. We demonstrate that the conformation of FN, regardless of the amount of FN adsorbed on materials surfaces, is *per se* a factor to trigger cell migration.

## Supplementary Material

Supplementary movie

Supplementary movie

Supplementary information
