# Supplementary material for: Cell migration on material-driven fibronectin microenvironments
Source: Biomater Sci. 2017 Jun 6;5(7):1326–33. doi: 10.1039/c7bm00333a (PMC5858633; doi:10.1039/c7bm00333a)
Supplement: Supplementary file 3 [file BM-005-C7BM00333A-s003.pdf]

Electronic Supplementary Material (ESI) for Biomaterials Science.
This journal is © The Royal Society of Chemistry 2017

Cell migration on material-driven fibronectin microenvironments

Eleni Grigoriou,[1] Marco Cantini,[1] Matthew J. Dalby,[2] Ansgar Petersen,[3]* Manuel Salmeron-Sanchez[1]*

[1]Division of Biomedical Engineering, School of Engineering, University of Glasgow, Glasgow, UK

[2]Centre for Cell Engineering, University of Glasgow, UK

[3]Berlin Brandenburg Center for Regenerative Therapies, Charité-Universitätsmedizin Berlin, Berlin, Germany

* Corresponding authors
Manuel Salmeron-Sanchez, Manuel.Salmeron-Sanchez@glasgow.ac.uk
Ansgar Petersen, Ansgar.Petersen@charite.de

Supplementary material

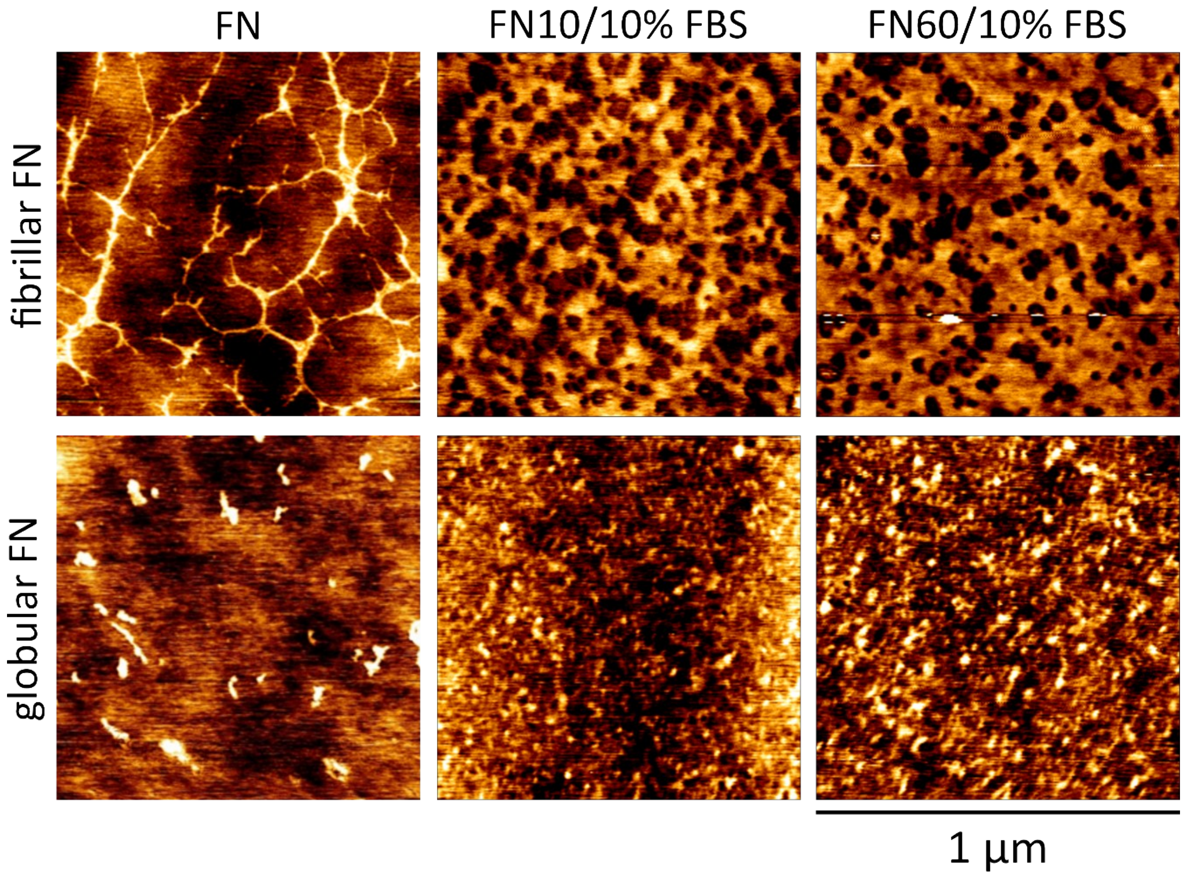

Figure S1. Height AFM images of PEA and PMA after FN adsorption for 10 min (left), FN adsorption for 10 min followed by incubation in DMEM supplemented with 10% FBS for 1 h (middle) and FN adsorption for 1 h followed by incubation in DMEM supplemented with 10% FBS for 1 h (right). The fibrillar and the globular conformation of the adsorbed protein layer is maintained after FBS adsorption on PEA and PMA respectively.

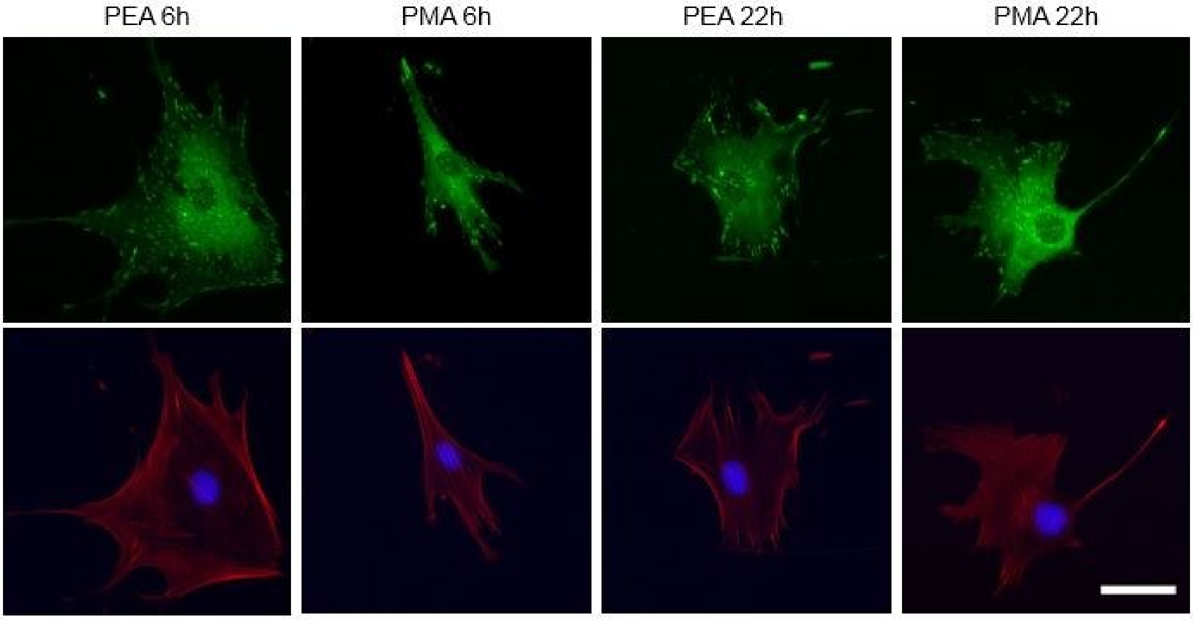

Figure S2. Focal adhesion formation of human fibroblasts on fibrillary and globular FN 6 h and 22 h after seeding. Fluorescent staining of focal adhesion (green), actin cytoskeleton (red) and nuclei (blue). Scale bar: 50 µm.

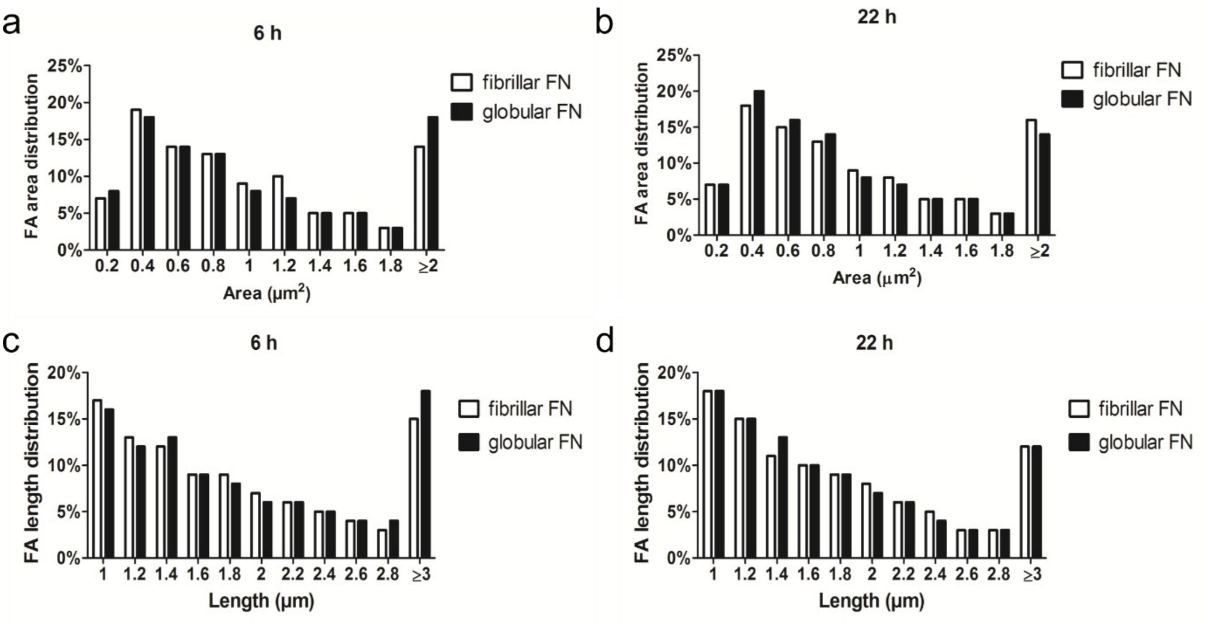

Figure S3. Area and length distribution of focal adhesions of human fibroblasts. Comparison of fibrillar and globular FN 6 h (a and c) and 22 h (b and d) after seeding.

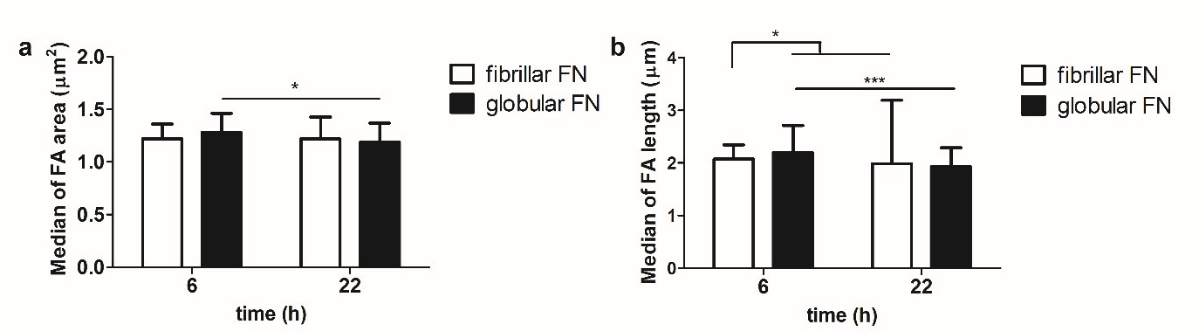

Figure S4. Median of focal adhesion area (a) and focal adhesion length (b) of human fibroblasts on fibrillar and globular FN (white and black bars respectively) 6 h and 22 h after seeding.

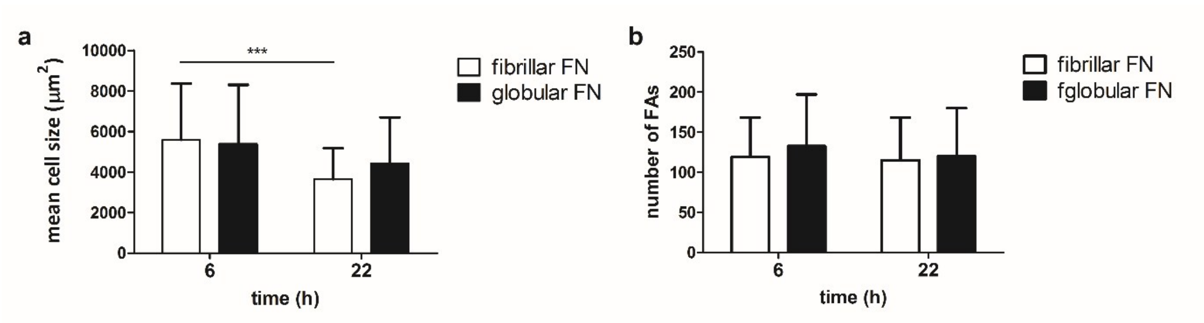

Figure S5. Average size of human fibroblasts on fibrillar and globular FN 6 h and 22 h after seeding (a). Average number of focal adhesions formed in human fibroblasts on fibrillar and globular FN 6 h and 22 h after seeding (b).

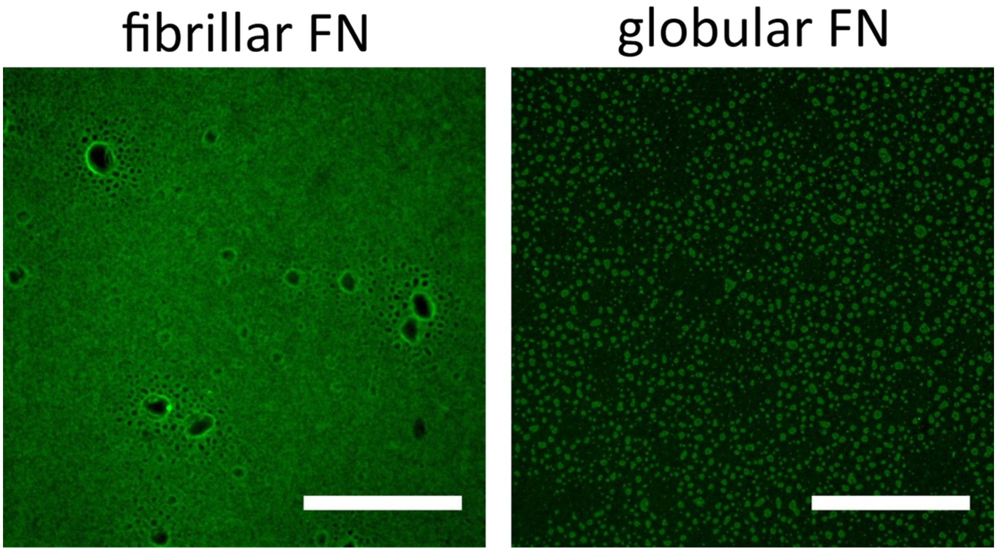

Figure S6. PEA and PMA samples were coated with FITC-FN for 1 h and incubated in culture medium. Reorganisation of the FN layer was not observed in the absence of cells. Scale bar is 40 μm.
